# Structural MRI Brain Alterations in Borderline Personality Disorder and Bipolar Disorder

**DOI:** 10.7759/cureus.16425

**Published:** 2021-07-16

**Authors:** Jack B Ding, Kevin Hu

**Affiliations:** 1 Psychiatry, Royal Adelaide Hospital, Adelaide, AUS; 2 Psychiatry, University of Adelaide, Adelaide, AUS; 3 Radiology, Lyell McEwin Hospital, Adelaide, AUS

**Keywords:** borderline personality disorder, bipolar disorder, structural mri, neuroradiology, neuropsychiatry, structural imaging

## Abstract

Bipolar disorder (BD) and borderline personality disorder (BPD) share many behavioral features, such as periods of marked affective lability and instability. Although there is a symptomatic overlap, the two disorders may be differentiated based on longitudinal course, phenomenology, and treatment responsiveness. In addition, the emotional changes in BPD are generally influenced by interpersonal factors, whereas BD episodes tend to be more sustained. We performed a literature review on the structural MRI features of both disorders and compared the findings. There are differences in areas of white and gray matter volumes and thickness in BP and BPD. BPD primarily affects the fronto-limbic network, in particular, the amygdala, hippocampus, and orbitofrontal cortex, whereas BP affects both cortical and subcortical areas. There are a limited number of large studies, and many studies examined in this review did not adjust for confounding factors or motion artifacts, which limit the utility of current data.

## Introduction and background

Ever since early studies demonstrated an association between ventriculomegaly and schizophrenia, neuroimaging has been seen as a potentially influential tool in diagnostic psychiatry [1]. The fundamental drive behind psychoradiology has been conceptualized as providing an interface between traditional phenomenological constructs and behavior and the underlying biology of psychiatric illnesses [2]. That is, neuroimaging has the potential to assist a clinician in delving closer to the organic source of the psychopathology as opposed to using behavioral factors alone.

Borderline personality disorder (BPD) is a complex mental disorder characterized by profound difficulties in regulating emotions, impulsivity, self-harming and suicidal behavior, interpersonal instability, and an intense fear of abandonment [3]. BPD has been heavily investigated with structural and functional MRI studies over the previous decades to detect possible organic changes underpinning the emotionally intense behavioral manifestations [4].

**Figure 1:**
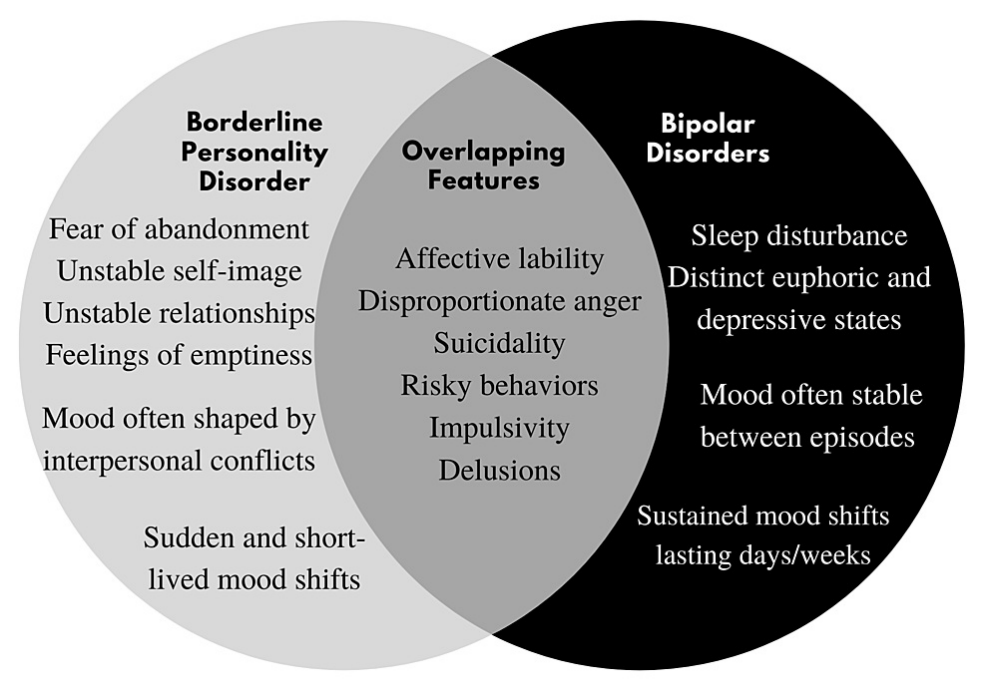
A comparison of signs and symptoms of borderline personality disorder and bipolar disorder.

Bipolar disorder (BD) is a serious mental disorder characterized by episodic affective instability, with the disease course fluctuating between depression, mania, and hypomania [2]. As shown in Figure 1, patients with BPD and BD can exhibit significant overlap in symptomatology.

Structural and functional neuroimaging have been used to investigate BD for several decades to assist an earlier diagnosis of BD and/or reveal tangible changes that may correlate with its behavioral symptoms [2]. A clinical diagnosis of BD is frequently delayed, with some studies reporting the time between patient presentation and formal diagnosis being over 10 years [5,6]. Around 69% of patients with BD are initially erroneously diagnosed with unipolar depression [5]. The misdiagnosis of BD initially presenting as depression is particularly clinically significant due to a subsequent lack of mood-stabilizing medications for the patient, as well as the potential risk of deteriorating to rapid cycling [7].

Both bipolar spectrum disorders and BPD are common psychiatric disorders, with the former having a lifetime prevalence of 2.4% and the latter between 3% and 5.9% [8-10]. The percentage of BD patients with a concomitant diagnosis of BPD has been reported to be as high as 16% [11]. Because both BPD and BD can manifest as periods of profound affective lability and instability, the interface between the two disorders has been hotly debated in the past, with some authors proposing that they should be classified as part of the same disease spectrum, as opposed to being distinct disorders [12]. Whether BPD should be a part of the bipolar spectrum or if it should be characterized as such is still debated, albeit most err towards the notion that they are distinct disease entities.

In terms of correlating neuroanatomical alterations to BPD and BD, the limbic regions are naturally of the foremost interest. The amygdalas are two nuclei clusters located medial to the temporal lobes and are considered to be a component of the limbic system. Amygdala is thought to play the primary role of regulating emotional responses, memory processing, and decision-making [13]. The hippocampi are located in the allocortex and project to the neocortex and are thought to be involved in memory consolidation and spatial memory [14].

In this review, we focus on the structural MRI brain changes seen in patients with BD and BPD. We then compare and contrast the structural changes seen in BD and BPD.

## Review

Methodology

Using a systematic process, a search of papers catalogued in PubMed was done in June 2021. The following search terms were used to identify articles pertaining to BD: “MRI” OR “structural imaging” AND “manic-depressed” or “manic depression” or “bipolar.” A second search was then performed to identify articles pertaining to BPD: “MRI” OR “structural imaging” AND “borderline personality” OR “BPD.” The abstracts and reference lists of each article were manually reviewed to assess their relevance. Studies were selected based on the following inclusion criteria: (1) human-only papers, (2) publication dates within the last 10 years, and (3) papers published in the English language. Studies were excluded based on the following exclusion criteria: (1) papers that used animals, (2) papers published over 10 years ago, (3) papers not published in English, and (4) papers studying functional MRI series.

The initial search yielded 726 articles. Table 1 identifies the search strategy and initial results. After applying the inclusion and exclusion criteria and removing duplicate studies, we identified 20 articles for further review.

**Table 1:** Summary of search strategy and initial search results.

Keywords	Database	Date of search	Number of results
(“MRI” OR “structural imaging”) AND (“manic-depression” OR “manic-depressed” OR “bipolar”)	PubMed	3/6/2021	636
(“MRI” OR “structural imaging”) AND (“borderline personality” OR “BPD”)	PubMed	3/6/2021	90

Table 2 provides an overview of the various aspects of the studies that met the criteria for inclusion in this review. Most papers used the Diagnostic and Statistical Manual of Mental Disorders-IV criteria and were volumetric-based studies. There were only two studies that directly investigated and compared structural MRI changes in BPD and BD patients using the same study design.

**Table 2:** An overview of the design and findings of the studies analyzed in this review. VBM: Voxel-based morphometry analysis; SPM: statistical parametric mapping; SBM: source-based morphometry; GM: gray matter; WM: white matter; HC: healthy control; MDD: major depressive disorder; CC: clinical control; SCZ: schizophrenia; SCZA: schizoaffective; DSM: Diagnostic and Statistical Manual of Mental Disorders

Author (s)	Study design	BPD patients	BD patients	Controls	Criteria	Results
Rossi et al., 2012 [15]	1.5T MRI, manual tracing of hippocampi, SPM processing	26	15	41	DSM-IV	Patients with BPD exhibited decreased volumes of the bilateral hippocampi, whereas BD patients displayed reductions in the right hippocampus volume
Depping et al., 2016 [16]	SBM to identify GM volume variation	17	0	22 (HC), 22 (MDD)	DSM-IV	Decreased GM volume was seen in the medial temporal/medial frontal network, cingulate, and lateral prefrontal regions of BPD patients compared to the HC group
Richter et al., 2014 [17]	Cortical thickness analysis and MRI measures of differences in hippocampal and amygdalar volume using FreeSurfer software	20	0	20 (HC), 20 (CC)	DSM-IV	Decreased volume in frontal and parietal regions in adolescent BPD participants was observed versus controls. There was no significant differences in cortical thickness
Schienle et al., 2015 [18]	Voxel-based morphometry to measure GMV in amygdala; changes correlated to self-reported symptom severity	25	0	25	DSM-V	Patients with BPD exhibited increased GM volume in the laterobasal amygdala. The GM volume of the centromedial amygdala was negatively correlated with self-reported symptoms
Soloff et al., 2012 [19]	VBM with SPM to differentiate BPD suicide attempters versus non attempters	68	0	52	DSM-III and DSM-IV	Decreased GM density was observed in BPD patients who had a history of suicide attempts compared to the control group and the nonsuicide attempter group
Jin et al., 2016 [20]	VBM to assess differences in GMV and GMC	34	0	34	DSM-IV	BPD participants showed increased GM volume in the middle cingulate cortex, posterior cingulate cortex, and precuneus
Araujo et al., 2014 [21]	1.5T MRI, FreeSurfer analysis of cortical morphology and volume	25	0	25	DSM-IV	There were substantial cortical morphologic changes in BPD patients versus controls
De Araujo Filho et al., 2014 [22]	1.5T MRI, Freesurfer software	25	0	25	DSM-IV	Patients with BPD had decreased cortical thickness and curvature in the left and right medial orbitofrontal cortex
Depping et al., 2018 [23]	3T MRI to measure local gyrification index	17	0	22 (HC), 22 (MDD)	DSM-IV	Decreased cortical folding of parahippocampal gyrus, precuneus, and superior parietal gyrus was seen in BPD patients
Rossi et al., 2015 [24]	1.5T MRI, GMD studied with cortical pattern mapping	26	0	16	DSM-IV	Areas of GM abnormality correspond to the regions responsible for the psychological characteristics of patients with BPD
Hibar et al., 2018 [25]	Both 1.5T and 3T MRI, with Freesurfer processing	0	1,837	2,582	Multiple diagnostic criteria	There was cortical GM thinning in frontal, temporal, and parietal regions bilaterally, with the greatest thinning at left pars opercularis, left fusiform gyrus, and left rostral middle frontal cortex. Notably, a longer duration of illness was associated with greater cortical thinning, and antipsychotic treatment was associated with changes in cortical thickness
Achalia et al., 2020 [26]	Structural MRI, FreeSufer processing	0	30	30	DSM-IV	Patients with BD with multiple manic episodes had lower cortical measures than those with a single manic episode
Knöchel et al., 2016 [27]	3T MRI, FreeSurfer processing	0	34	32 (SZ), 38 (HC)	DSM-IV	Patients with BD exhibited cortical thinning in the pars operculate of the inferior frontal gyrus bilaterally and the anterior and posterior cingulate bilaterally
Li et al., 2018 [28]	3T MRI, FreeSurfer processing of cortical reconstruction and volumetric segmentation of the whole brain	0	35 BD	30	DSM-IV	There was widespread thinning of the prefrontal cortex in patients with BD. Patients on antipsychotic treatment showed significantly increased cortical thickness
Hibar et al., 2016 [29]	Both 1.5T and 3T MRI, with FreeSurfer processing	0	1,710	2,594	Multiple diagnostic criteria	There were volumetric reductions in the hippocampus, thalamus, as well as enlarged lateral ventricles
Rossi et al., 2013 [30]	VBM with SPM to identify and measure GM and WM changes	26	14	40	DSM-IV	Cortical and subcortical alterations were more specific to BD, whereas fronto-limbic changes were more specific to BPD. Both BPD and BD patients exhibited GM density changes, though the changes were more severe in BD
Kim et al., 2020 [31]	3T MRI with cerebellum segmentation processing of cortical regional thickness and cerebellum volume	0	90	166	DSM-IV	There was widespread and significant cortical thickening in all 12 cerebellar subregions
Abé et al., 2016 [32]	3T MRI, FreeSurfer processing	0	81 with BDI, 59 with BD II	85	DSM-IV	BDI and BDII participants both showed reduced cortical volumes, thickness, and surface area. BDI showed reduced volume and thickness in temporal and medial prefrontal regions
Zhao et al., 2021 [33]	3T MRI, VBM8 toolbox processing	0	70 (nonsuicide attempters), 40 (suicide attempters)	110	DSM-IV	Bipolar patients with a history of suicide attempts had lower gray matter volume in the medial prefrontal cortex, ventral prefrontal cortex, and dorsolateral prefrontal cortex compared to those without a history of suicide attempts
Jiang et al., 2021 [34]	3T MRI, VBM toolbox processing	0	15	20 (MDD), 30 (HC)	DSM-IV	BD patients showed decreased GM volume versus MDD and HC in the supramarginal gyrus, the superior temporal gyrus, the Rolandic operculum, the postcentral gyrus, the insula, the superior parietal gyrus, the inferior parietal gyrus, the Heschl’s gyrus, and the angular gyrus in the right-sided cerebrum

Neuroanatomical features and neurobiology of borderline personality disorder

Regarding neuroanatomical alterations in BPD, regions of particular interest are the limbic structures, specifically the hippocampus and amygdala, given the substantial role the former plays in episodic memory and the latter in emotion regulation and modulation of the fear response [13,14]. Numerous earlier volumetric studies have investigated volume alterations in the limbic and paralimbic regions by manual tracing methodology and demonstrated that decreased volume in the amygdala and hippocampus is associated with BPD compared to healthy controls [15]. While these early studies were limited by potential confounders such as variable psychotropic treatment and psychiatric comorbidities, such as major depressive disorder (MDD), substance use disorder, and especially posttraumatic stress disorder (PTSD), a recent meta-analysis of the amygdala and hippocampal volume alterations in BPD concluded that these potential confounders were unable to completely account for the volumetric reductions of the amygdala and hippocampus in patients with BPD [35].

More recent structural imaging studies have adopted voxel-based morphometry (VBM) techniques to overcome some of the limitations of manual tracing. By adopting a VBM approach, Depping et al. showed that BPD patients had reduced volumes in the amygdala, hippocampus, parahippocampus, and medial frontal regions compared to patients with MDD and healthy controls [16]. Niedtfeld et al. used VBM techniques to investigate BPD patients with and without concomitant PTSD, and discovered that BPD symptom severity was negatively correlated with amygdala volume irrespective of the presence of PTSD [36]. A study involving 60 adolescents newly diagnosed with BPD employed VBM techniques and discovered a decrease in the right amygdala volume and hippocampus volume bilaterally in adolescents with BPD compared to the control group [17]. This study design is particularly significant as recent-onset BPD diagnosis in an adolescent age group ensured that long-term confounding variables such as medication or comorbidities did not play a substantial role in volumetric alterations of the limbic structures, implying that the changes were more likely attributable to BPD. These findings are in line with the conclusions of two recent meta-analyses, which concluded that BPD patients show volume reductions in limbic and paralimbic structures [35,37].

With the volumetric reduction in the amygdala being more definitively associated with BPD, one study investigated amygdala subdivisions and their correlation with self-reported symptom severity. Schienle et al. compared structural MRI data from 25 BPD participants with 25 controls using VBM techniques [18]. They found that BPD patients had greater left lateral basal amygdala gray matter volumes compared to controls which were positively correlated with symptom severity. In contrast, the centromedial amygdala volume was negatively correlated with symptom severity. Notably, the laterobasal amygdala is thought to play a primary role in decoding affective expressions and fear conditioning, whereas the centromedial amygdala functions to generate emotional arousal and behavioral responses [38,39].

It is worth noting that an inverse relationship between amygdala volume and disease is not unique to BPD. For example, a previous study showed that selective serotonin reuptake inhibitors (SSRIs) normalized low amygdala volumes after a trial of SSRIs in patients with MDD [40].

In addition to volumetric reductions of limbic structures, studies have also investigated specific structural brain abnormalities in BPD patients and their relationship with psychological metrics. Soloff et al. demonstrated that suicide attempters with BPD had decreased gray matter in the left insula compared to nonattemptors with BPD and that several structural differences could discriminate high-lethality suicide attempts from low-lethality ones [19]. Jin et al. investigated 34 BPD participants and 34 controls using VBM and correlated their findings with childhood trauma questionnaires (CTQ) and attachment style questionnaires (ATQ) [20]. They found that BPD patients demonstrated gray matter volume increase in the precuneus and middle/posterior cingulate cortices. CTQ and ASQ scores were not correlated with gray matter volume in any region for BPD patients.

Furthermore, numerous studies have investigated cortical changes in BPD patients. Araujo et al. compared several morphometric and geometric parameters of each hemisphere and reported that BPD patients demonstrated significant changes in the fronto-limbic regions compared to controls [22]. This is congruent with previous studies that have suggested that the orbitofrontal cortex (OFC) and anterior cingulate cortices are involved in BPD pathophysiology [41].

Filho et al. used a surface-based processing approach demonstrating that patients with BPD had reductions in cortical thickness and curvature in the left medial OFC and right medial OFCs compared to controls [22]. A novel discovery was extensive structural differences in the bilateral medial OFCs in patients with BPD who had possible hemispheric asymmetry. Depping et al. used structural MRI to measure the gyrification index, a metric of cortical folding and neurodevelopment, and discovered diminished cortical folding of the parahippocampus, precuneus, and superior parietal gyrus in BPD patients compared to controls [23]. Notably, they found a negative association between the local gyrification index of the orbitofrontal regions and impulsivity in patients with BPD. While the local gyrification index differences are not specific to BPD, this revealed that aberrant early neurodevelopment may underpin BPD pathophysiology.

Rossi et al. measured cortical gray matter density in patients with BPD versus controls with MRIcro and SPM software processing [24]. They found that BPD patients had lower gray matter density in the bilateral superior temporal gyri, inferior and middle frontal gyri, dorsal frontal cortex, anterior/posterior cingulate cortices, and temporal lobe on the lateral and medial left cortex. Conversely, there was increased gray matter density in the sensory-motor areas and right superior frontal gyrus. Notably, these findings are localized in cortical areas that underpin psychological functions that are known to be deficient or deregulated in patients with BPD [42].

To summarize thus far, components that make up the fronto-limbic network, specifically the amygdala, hippocampus, OFC, and deep prepiriform cortex are thought to be implicated in the pathophysiology of BPD and thus have been investigated initially with the region of interest followed by VBM techniques. The structural differences detected by earlier studies were potentially confounded by psychiatric comorbidities, especially PTSD, and remained unaccounted in medication management. However, numerous meta-analyses and studies investigating these confounders suggested that fronto-limbic network alterations were present [37,43]. One potential confounder that was not adjusted for by any study may involve the general inclusion of people with BPD. Given the heterogeneity of BPD, there can be vast differences in symptoms among patients diagnosed with BPD [44]. Therefore, it is not unreasonable to suggest that a patient with BPD who is predominantly dissociative or psychotic may have different neurological alterations compared to a patient whose main clinical complaint is impulsiveness.

Neuroanatomical features and neurobiology of bipolar disorder

In terms of investigating volume and thickness modifications in BD, Hibar et al. performed the largest study to date, analyzing MRI scans of 6,503 individuals with or without BD [25]. In BD, the cortical gray matter was thinner in frontal, temporal, and parietal regions of both brain hemispheres. Patients with a longer history of diagnosis of BD had reduced cortical thickness in frontal, medial parietal, and occipital regions. Achalia et al. demonstrated that structural changes seen in BD, such as loss of cortical volume and surface area, are more pronounced in patients who experience multiple manic episodes compared to a single episode [26]. However, these results may be confounded among patients with multi-episode BD who are more likely to have been treated by various combinations of mood stabilizers and antipsychotics. Additionally, appropriate therapy for BD with antipsychotics affects cortical thickness. Knochel et al. demonstrated thinning in the pars opercularis in the inferior frontal gyrus bilaterally and both the anterior and posterior cingulate bilaterally. This frontal thinning is associated clinically with deficits in psychomotor speed and executive functioning. This study additionally provided a link between neuroanatomical features and clinical severity of BD [27]. Li et al. demonstrated reduced cortical thinning in the prefrontal cortex in patients with BD on lithium or valproate medication compared to healthy controls [28]. This study was limited by its cross-sectional design and was confounded by inconsistent medication treatment, with some patients being on several different antidepressant or anxiolytics which may interact. Despite these limitations, these studies suggest that the pathophysiological processes in BD may result in progressive neuroanatomical changes which may be influenced by treatment. This may assist in determining neuroimaging biomarkers for BD.

Hibar et al. examined subcortical structures in BD patients and found significant subcortical volumetric reductions in the hippocampus, thalamus, and lateral ventricles [29]. Rossi et al. examined MRI images of 26 patients with BPD and 15 patients with BD and found that BD and BPD patients share features of reduced hippocampal volumes, with the bipolar group showing significantly reduced right hippocampal volumes, and the BPD group showing smaller hippocampal volume bilaterally [30].

The cerebellum has been implicated not only in motor control but also higher-order cognitive and emotional functions [44]. However, previous studies have been highly variable regarding cerebellar findings. While the study by Rossi et al. [30]. reported cerebellar volume loss, along with volume loss in other cortical structures, Laidi et al. suggested that decreased cerebellar cortical volume is specific to schizophrenia [35] and that cerebellar volume changes in BD are more subtle if present. These findings were further confounded by a cross-sectional study by Kim et al. [31] who found widespread and significant cortical thickening in all 12 cerebellar subregions, as well as reduced cerebellar volumes in left lobule IX in BD patients. Another small pilot study could not identify volumetric changes in the cerebellum [45].

Between BD subtypes, namely, BD1 and BDII, a study of 81 patients with BDI and 59 patients with BDII found reduced cortical volume and thickness in the temporal and medial prefrontal region, whereas, in BDII, this was preserved [32]. These findings conflicted with a larger study of 1,710 patients with BD, which concluded there were no significant differences in brain volume between BDI and BDII patients [29]. Both studies did not account for premorbid conditions affecting the group differences, for example, social, environmental, or genetic factors.

Zhao et al. examined BD patients with and without suicidal attempts. They showed that patients who had attempted suicide had reduced gray matter volumes in various areas of the prefrontal cortex area compared to BD patients without suicidal attempts [33]. This is consistent with previous studies that also demonstrated changes in various areas including the prefrontal cortex [46].

Contrasting the neuroanatomical alterations of borderline personality disorder and bipolar disorder

Out of the studies examined, only two directly compared structural differences between BP and BPD [15,30]. The earlier 2012 study revealed that both BPD and BD patients had reduced hippocampal volumes, with BD reductions being localized to the right hippocampus [15]. The 2013 study used VBM techniques and focused on measuring gray matter and white matter changes in BP and BPD patients [30]. The BD participants displayed wide regions of gray matter loss in the cerebellum and thalamus compared to healthy individuals, whereas the fronto-limbic network was more specific to patients with BPD. These findings were supported by another study with a similar design, which demonstrated reduced gray matter volume in various areas of the parietal and occipital lobe bilaterally in both MDD and BD patients [34].

Other studies examined in this review suggested that patients with BPD have decreased amygdala volumes compared to healthy controls, whereas amygdala volumes are normal in BD patients [17,18]. The amygdala is known to play a crucial role in emotional regulation, and previous functional neuroradiology studies have suggested a link between amygdala hyperactivation and emotional dysregulation in BP [47]. Because BD can present with similar affective lability, the emotional changes in BPD and BD cannot be explained by the structural alterations of the amygdala alone. The functional and structural modifications of the amygdala in BD and BPD are worth further investigation.

Structural imaging studies of BD identified decreased volumes particularly in the thalamic and hippocampal regions [15,25,30]. Of these changes, volumetric alterations in the thalamus seemed to be specific to BD, whereas BPD patients exhibited volumetric reductions in the hippocampi. Although one should not be inclined to suggest clinical manifestations based only on alterations in brain volume, the fact that both conditions exhibit similar structural changes to a limbic component speaks volumes regarding possible underlying pathophysiology. In addition, these findings are limited by sample size, with only one large-scale study investigating those structural changes in BD patients. Given widespread conflicting findings in other studies investigating structural changes in BD, further studies comparing structural modifications of the thalamus and hippocampus in BD and BPD are recommended. The abovementioned neuroanatomical findings are listed in Table 3.

**Table 3:** Neuroanatomical alterations of BPD and BD noted on structural MRI. BPD: borderline personality disorder; BD: bipolar disorder; GM: gray matter

BPD-specific changes	Overlapping changes	BD-specific changes
Amygdala volume reduction [17,18]	Hippocampal volume reduction [15]	Cerebellum GM loss [30]
		Thalamus GM loss [30]
		Parietal and occipital GM loss [34]

## Conclusions

Imaging studies performed suggest that BD and BPD are unlikely to localize to abnormalities within single, discrete neuroanatomic structures. Instead, there is a range of changes within complex neural networks. In terms of findings specific to each disorder, the studies we investigated suggested smaller amygdala volume in BPD patients compared to healthy controls, whereas thalamic volumetric alterations were only seen in BD patients. In terms of overlap, studies reported hippocampal volumetric reductions and gray matter and white matter reductions in both BD and BPD. Several studies identified relationships between structural brain abnormalities and various psychological metrics, for example, changes in the left insula associated with suicide attempts in BPD and reduced cortical thickness in BD patients with multiple manic episodes. This suggests a correlation between disease progression and structural changes in both BPD and BD.

There was a scarcity of large-scale studies that adjusted for confounding factors such as psychiatric comorbidities, medication management, or technical factors such as motion artifact. Future head-to-head comparisons with other psychiatric disorders such as PTSD may be of benefit. Further direct structural imaging comparisons between BPD and BD further exploring thalamic involvement or the relationship between structural imaging findings and various psychological markers may also be of value.
